# Explicit modeling of antibody levels for infectious disease simulations in the context of SARS-CoV-2

**DOI:** 10.1016/j.isci.2023.107554

**Published:** 2023-08-08

**Authors:** Sebastian A. Müller, Sydney Paltra, Jakob Rehmann, Kai Nagel, Tim O.F. Conrad

**Affiliations:** 1 Technische Universität Berlin, FG Verkehrssystemplanung und Verkehrstelematik, 10623 Berlin, Germany; 2 Zuse Institute Berlin, 14195 Berlin, Germany

**Keywords:** Health sciences, Immunology, Virology

## Abstract

Measurable levels of immunoglobulin G antibodies develop after infections with and vaccinations against severe acute respiratory syndrome coronavirus 2 (SARS-CoV-2). These antibody levels are dynamic: due to waning, antibody levels will drop over time. During the COVID-19 pandemic, multiple models predicting infection dynamics were used by policymakers to support the planning of public health policies. Explicitly integrating antibody and waning effects into the models is crucial for reliable calculations of individual infection risk. However, only few approaches have been suggested that explicitly treat these effects. This paper presents a methodology that explicitly models antibody levels and the resulting protection against infection for individuals within an agent-based model. The model was developed in response to the complexity of different immunization sequences and types and is based on neutralization titer studies. This approach allows complex population studies with explicit antibody and waning effects. We demonstrate the usefulness of our model in two use cases.

## Highlights

• Development of a new model to simulate antibody levels of individual agents
• Modeling of variant-specific antibody levels in the context of SARS-CoV-2
• Ability to handle immune escape variants based on antibody titer measurements
• Calculation of variant-, age-, and time-dependent protection against infection

## Graphical abstract

**Figure:**
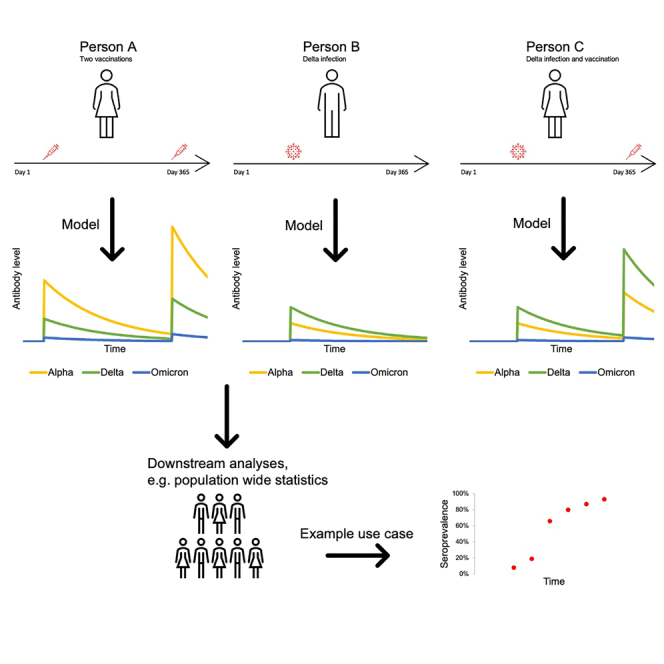

## Introduction

Measurable immunoglobulin G (IgG) antibodies to *severe acute respiratory syndrome coronavirus 2* (SARS-CoV-2) antigens develop after most infections with and vaccinations against SARS-CoV-2.^1^ Although the extent of immunity associated with different antibody titers and other immune responses is not yet fully understood, it is highly likely that an individual’s antibody level provides some information about their specific risk and severity of a future infection.^2^^,^^3^ However, SARS-CoV-2 IgG antibody levels decrease over time if no further immunization event occurs.^4^ This waning process has been confirmed in multiple studies, showing similar effects regardless whether the immunization happened through vaccination or infection.^5^^,^^6^ It has been consistently shown that the total antibody level starts declining about six weeks after the immunization event and potentially reduces by more than 50% over 10 weeks.^7^^,^^8^^,^^9^ Hence, waning is important and should be considered explicitly when modeling the antibody level.

During the COVID-19 pandemic, multiple models for projecting and predicting the spread of infections have been developed. In many countries, researchers and policymakers have been using these models to simulate and implement public health policies. From a modeling perspective, explicitly integrating antibody and waning effects into the simulation framework is crucial to allow reliable calculations of the individual risk of infection and severeness estimation. So far, only very few approaches have been suggested that explicitly treat these effects (see Section Antibody Models for Epidemiological Predictive Modelling of COVID-19: A Literature Search).

In this paper, we describe how to model antibody levels explicitly on an individual level, such that the population-wide statistics are close to reality. This approach can be integrated into general frameworks, allowing complex population studies with explicit antibody and waning effects. We demonstrate the usefulness of our model in two use cases: First, we show how to model a population, based on available data, which allows the derivation of time-dependent immunization statistics of the individuals. Second, we describe how the antibody model can be used to calculate protection levels (against infection) from virus variants for the entire population, specific sub-groups or on the individual level.

The contributions of this paper are 3-fold.

1. We briefly review the current state-of-the-art literature concerning approaches for modeling individual antibody levels in epidemiological predictive models of COVID-19 (see Sec. Antibody Models for Epidemiological Predictive Modelling of COVID-19: A Literature Search).
2. We present an approach that uses antibody levels to model effects such as waning or differentiated protection against different variants based on different individual immunization sequences. This is based on available data, such as vaccination and infection statistics or neutralization titers. The use of neutralization titers allows simulations to be performed before vaccine effectiveness studies are available. This is very useful when new virus variants emerge and therefore one of the major advantages of the approach presented here.
3. We show how information about the antibody level from our model can be translated into an individual’s specific *protection levels* against different SARS-CoV-2 virus variants (wild-type, Delta, Omicron BA.1, Omicron BA.2, etc.).

## Results

### Model overview

**Figure 1:**
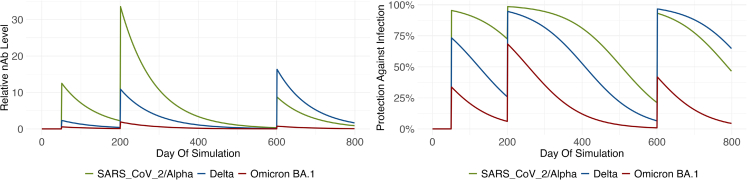
Exemplary immunization history The agent gets infected with the wild type on day 50, receives the mRNA vaccine on day 200, and gets infected with the Delta variant on day 600. Left: Neutralizing antibody levels, Right: Resulting protection against infection (Protection is computed as follows: 1−immFac=VE).

We propose a generalized antibody model. We assume generalized antibody levels, $N_{ab,v}$, against all virus variants *v* of interest. These generalized antibody levels are initially zero. The first immunization event, be this a vaccination or an infection, sets all $N_{ab,v}$ to initial levels, which need to be calibrated. From then on, all levels follow an exponential decay, until another immunization event pushes them up again (the principle is shown in Figure 1).

So far, this follows pre-existing models in the literature. The novelty is that we plug this generalized antibody model into a dose-response model for agent-based infection dynamics. Mathematically, the dose-response model uses an infection probability of

$$p_{\inf,v}=1-\exp\;\left(-\Theta_{v}\cdot d_{v}\right)\text{,}$$

where $d_{v}$ is the viral dose of the virus variant *v*, and $\Theta_{v}$ is a calibration parameter, which depends on the transmissibility of the virus variant under consideration. In order to calculate the reduced infection probability of immunized persons, we integrate generalized antibody levels in the model as follows:

(Equation 1) $$p_{\inf,v}^{immunized}=1-\exp\;\left(-\Theta_{v}\cdot \frac{d_{v}}{1+N_{v}^{\beta }}\right)\;\text{.}$$

Clearly, for $N_{v}=0$ the model returns to its previous form, and $N_{v}=1$ has the same effect as dividing the dose by 2.

### Calibration

**Figure 2:**
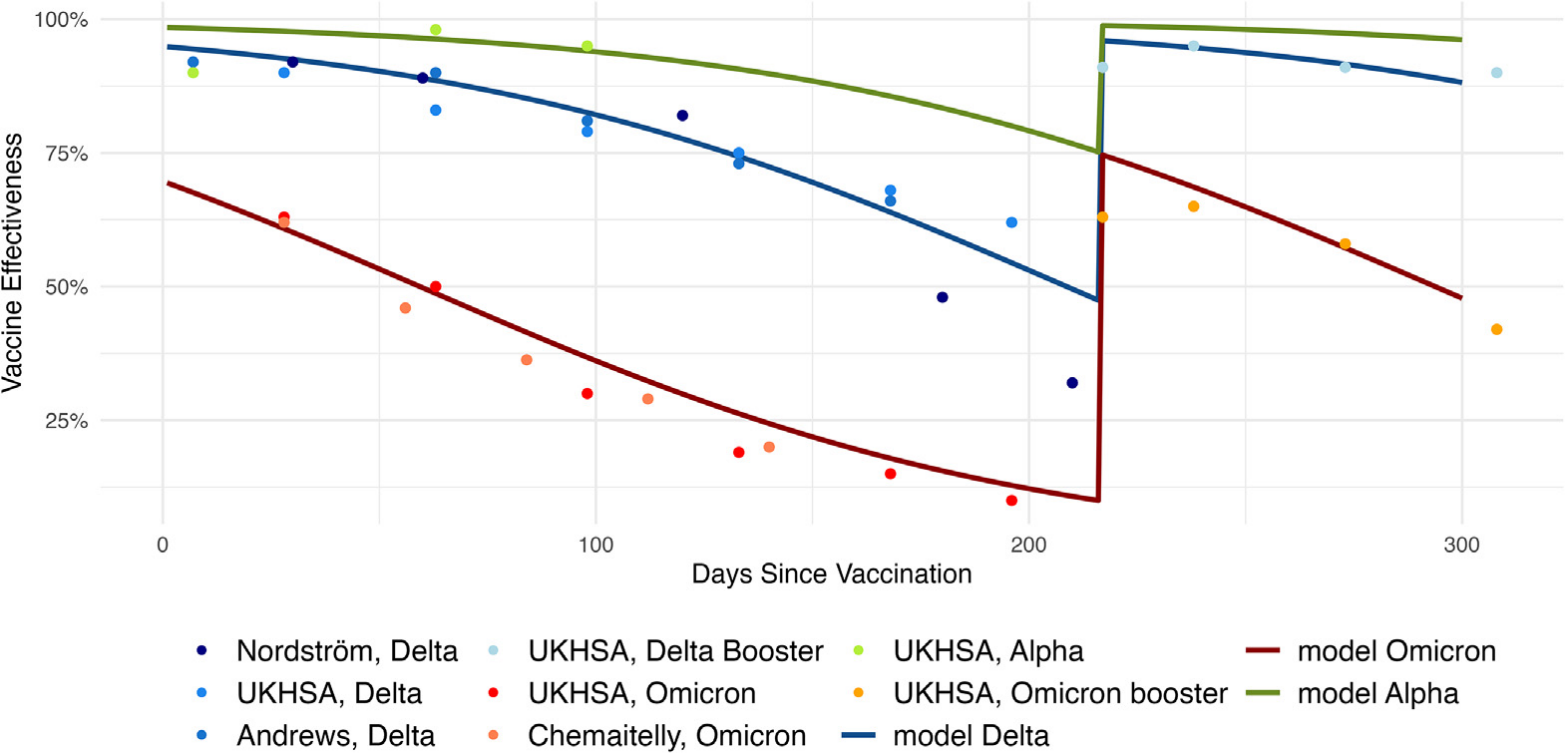
Calibration results. Dots were taken from the literature,^15^^,^^16^^,^^20^^,^^21^^,^^22^^,^^23^ lines are the fitted curves On day 210 the agent receive a booster dose, which increases their level of protection.

Our model uses data from studies regarding vaccine effectiveness, VE, as input (see also Figure 2). These studies compare persons with a certain immunization history, i.e., vaccination(s) or infection(s), with persons without that; VE is typically given as a function of time after the last of these immunization events. At the beginning of a pandemic, such studies are comparatively straightforward to conduct since only one virus variant is in circulation, and many immunologically naive persons are available for comparison. Later in the pandemic, it becomes harder to study VE as people have increasingly divergent immunization histories, which leads to inhomogeneous immunity profiles; additionally, there are fewer immunologically naive persons to compare with. Our model interpolates the results of vaccination effectiveness studies, which allows us to generalize these to arbitrary sequences of immunization events. Additionally, it is possible to extend immunity profiles, i.e., a synthetic person’s $N_{v}$ values, to a new virus variant by using the lab-based neutralization titer measurements. Specifically, if a neutralization titer of a new virus variant *w* is reduced by a factor of α against an older virus variant *v*, all new $N_{w}$ values are obtained as

$$N_{w}=\alpha \cdot N_{v}\;\text{.}$$

### Use cases

In the following, we show two use cases of our model (details about STAR Methods and data can be found in Sec. Method details). All results refer to the city of Cologne in Germany.

1. We show that the model is able to calculate population-wide immunization statistics at a given time, even for large populations, i.e., people living in a large city. This is the basis for more complex use cases, such as the following one. Our model is validated by the fact that its outputs closely match the observed data.
2. We show how the model can be used to gain insight into individual population groups and how they are protected against different virus strains. The model can be used as a data source to develop strategies, such as vaccination campaigns, and can compensate for missing data.

#### Use case 1: Population-wide immunization statistics

The model presented in this paper can be implemented as an extension of our agent-based model (ABM).^10^ In this way, we can calculate infection dynamics that are also (but not only) dependent on immune protection. We show in the following section how the model can be used to calculate the population-wide immunity at a given point in time. This allows us to evaluate the model in a real-world scenario by comparing it to available data for time periods in which data are available. Moreover, since the strategies of policymakers often depend on the existing immunity in the population, this is a relevant parameter.

In Table 1, we show how our model result compares to observed data.^11^ The observed data contain information on the percentage of study participants who tested positive for antibodies against the S antigen and for antibodies against the N antigen. Antibodies against the N antigen indicate that the participant had an infection in the past. Antibodies against the S antigen indicate that the participant has been vaccinated or infected in the past. Therefore, we compare the two quantities with the percentage of the population in the model that has had at least one infection and at least one infection or vaccination, respectively. The observed data stem from measurements across Germany in the period from June 2022 until September 2022; the model data refer to Cologne and July 2022. As the observed data are available in this way, the comparison is made for different age groups. The age group < 18 is not shown because we do not have the observational data to compare it to.

**Table 1:** Comparison of population-wide immunity according to observed data from^11^ (1st and 3rd row) and our model (2nd and 4th row)

	all	18–29	30–34	35–39	40–49	50–59	60–64	65–79	>79
S antigen^11^	96%	96%	94%	94%	94%	95%	95%	97%	99%
>1 vaccination/infection (model)	97%	96%	96%	96%	96%	96%	98%	98%	98%
N antigen^11^	44%	59%	54%	52%	50%	42%	37%	35%	28%
>1 infection (model)	52%	55%	53%	52%	52%	51%	50%	50%	50%

The observed data refer to Germany and the period from June to September 2022; the model refers to Cologne and July 2022.

When comparing the observed data in Table 1 to the model results, one finds the following. (1) The proportion of participants testing positive for antibodies against the S antigen fits very well with the model results. According to the data, in the adult population, the proportion of those testing positive is 96%; in the model, approximately 97% had at least one infection or vaccination. (2) The proportion of those with at least one infection in the model fits the data less well, with a value of 44% in the data vs. 52% in the model. The deviations at this point could presumably be explained by the fact that the Cologne model is being compared here with data from all of Germany or that the proportion of undetected infections in the model is somewhat too high. The model is compared to reported case numbers in reality, and in order to model the population-wide immunity correctly, it thus needs to make assumptions about under-reporting, i.e., infections that occurred in reality but were not reported. In addition, the study that we compared our results to could be associated with some inaccuracies; e.g., because the measurements have a detection limit, fewer infections are found than have actually occurred. (3) In terms of the proportion with at least one infection, both the model and the data show that this proportion decreases with increasing age. However, the spread in the data between the different age groups is greater in reality than in the model. It is worth noting that our model does not currently account for the age distribution within households, which could contribute to the discrepancy. Additionally, the mobile phone data we utilized to simulate behavioral changes throughout the pandemic are not age dependent, potentially overlooking the higher cautionary behavior exhibited by older individuals in reality. This absence of age-dependent behavioral patterns might explain the observed deviation in our model’s representation.

In addition to the data shown in Table 1, there are further surveys^12^^,^^13^ that attempted to determine what proportion of the population has had at least one infection. We only found data from summer 2022, but the available studies show results similar to our model: by then, about 35%–50% of the German population had been infected with COVID-19 at least once. Since the proportion of vaccinated individuals is higher in the studies than that in the general population, this value must be interpreted as a lower limit and can only be used as a rough guide. For this reason, and because the study results vary somewhat, we believe our model results are acceptable.

**Figure 3:**
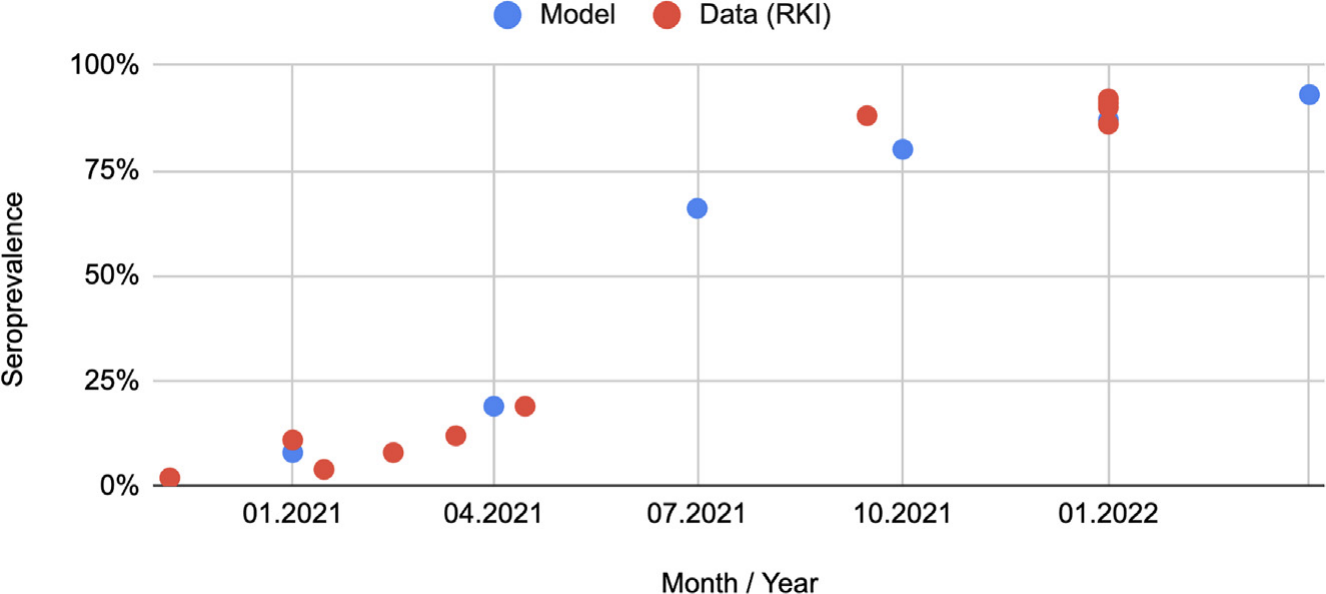
Comparison of seroprevalence in the model (blue) and observed data^14^ (red) over time The observed data refer to all of Germany, the model to Cologne.

The data shown in Table 1 allow a comparison to the model within the different age groups. In order to be able to compare the development over time, we use a publication from the RKI (Robert Koch-Institut), which has compiled various studies on seroprevalence in Germany. The nationwide results are shown in Figure 3 (red) and are compared with our model (blue). The model points show the proportion of the population with at least one infection or vaccination.

In general, it becomes clear that the model and the data match quite well here. At the beginning of 2021, there is a low seroprevalence in the population in both cases, which then increases significantly, especially in summer and fall 2021, and finally reaches values above 90%.

#### Use case 2: Variant-specific protection of sub-groups

In general, we assume in our calculations that there is no immune protection at the beginning of the pandemic and that each infection or vaccination increases protection, where protection is defined in the same way as vaccine effectiveness, i.e., a reduced probability to become infected compared with immunologically naive persons. The exact methodology is explained in Sec. Method details. In the following, we depict how the population is protected against infection from different virus variants according to the model presented in this paper. These are simulation results that are difficult to validate against real data because the real-world data are not available with this level of accuracy. The results must therefore be interpreted with some caution but offer considerable added value precisely because they cannot be collected in any other way. However, the results from the first use case show that where there are data to compare to, the model fits the data well when it comes to general immunity in the population.

**Figure 4:**
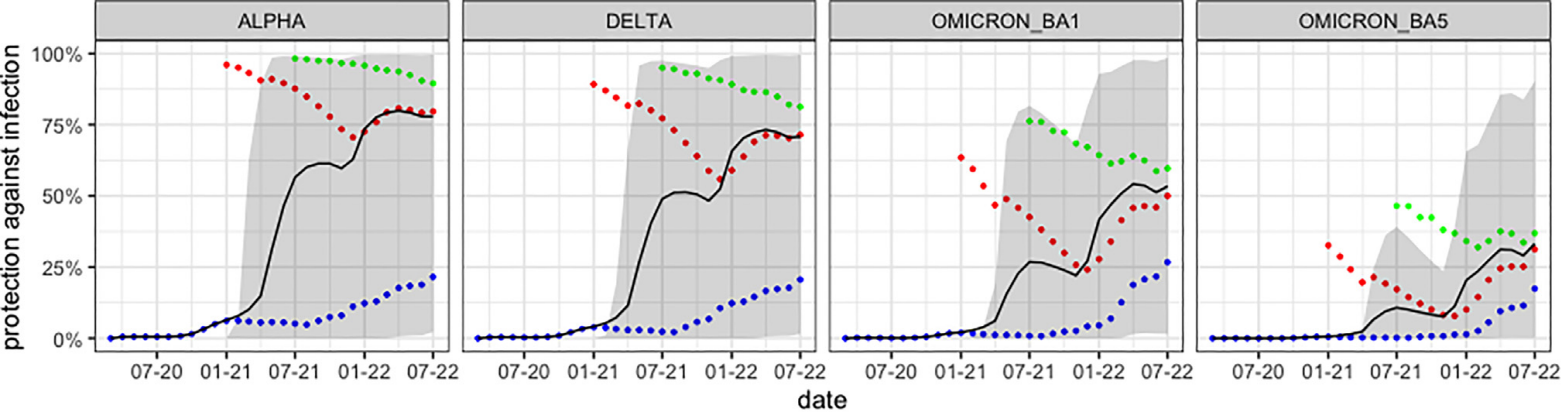
Protection against infection according to the model for different variants The color coding is as follows: blue: unvaccinated, red: vaccinated, green: boostered, black: mean protection (whole population), gray area: 10th to 90th percentile. The red dots (vaccinated) do not include boostered individuals, meaning that every person is part of only one group. Reading example: for the Delta variant (2nd plot) it becomes apparent that unvaccinated (blue) have a significantly lower protection that vaccinated (red) or boostered (green) by July 2022.

Figure 4 shows the population-wide protection against infection over time averaged over all age groups. The gray area shows that there is a large spread in immune responses; some individuals are subsequently very well protected, while others have almost no protection. This can partially be explained by the fact that some individuals are unvaccinated (blue dots), while others are vaccinated (red dots) or boostered (more than two vaccinations, green dots). The model results clearly show that vaccinated individuals are better protected than unvaccinated individuals, and missing vaccinations are not compensated for by infections. Thus, unvaccinated individuals do not achieve the same protection through infections as vaccinated individuals.

We calculate the protection for the different variants (left to right). Individuals do not have protection against any variants at the beginning of the pandemic and do not acquire significant immunity throughout 2020. This is because only a small fraction of the population was infected in 2020 and vaccinations were not yet available. Relevant immune protection is achieved by mid-2021 because vaccinations became available for the entire adult population. Beginning in July 2021, a significant decline in immune protection through waning is clearly visible. In winter 2021/2022, we see another protection increase when a third round of vaccinations (boosters) was administered to large segments of the population. The significant increase in immunity in the group of vaccinated persons (red dots) at the beginning of 2022 can be explained on the one hand by infections and on the other hand by the statistical effect that the group becomes smaller and smaller due to booster vaccinations. Due to the vaccination interval between the first vaccination and the booster vaccination, the proportion of persons in the group of vaccinated persons, for whom the vaccination took place a long time ago, is reduced more and more in the course of the booster campaign.

In addition, the different facets of Figure 4 show the impact of immune escape variants: in general, protection against infection with Alpha is significantly higher than that against Delta, and protection against Delta is significantly higher than that against either Omicron variant.

**Figure 5:**
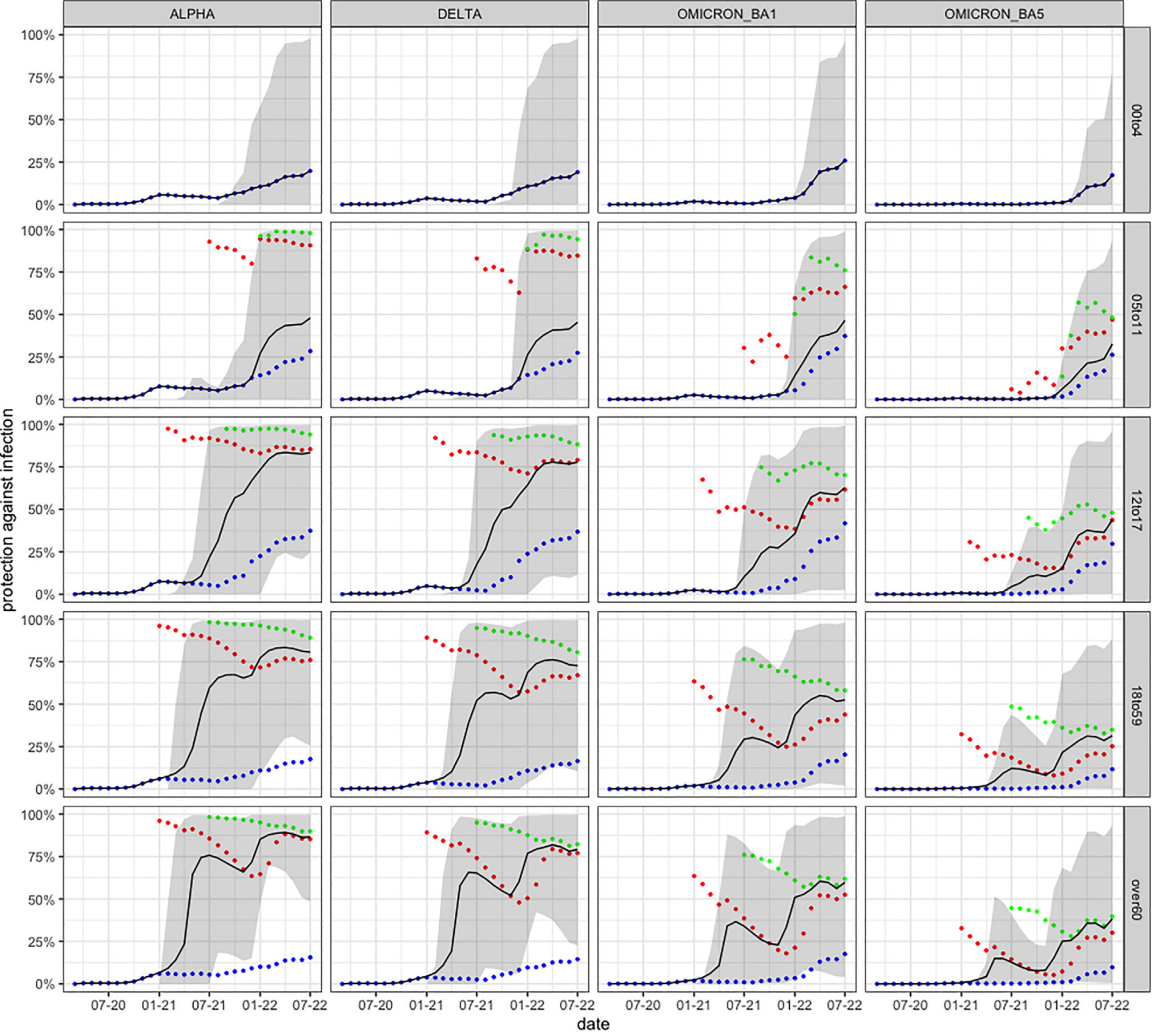
Protection against infection according to model for different variants and age groups The color coding is as follows: blue: unvaccinated, red: vaccinated, green: boostered, black: mean (whole age group).

Figure 5 shows how protection varies across age groups. It is clear that the mean protection between the age groups differs significantly. On average, children acquired less protection than adults, which can be explained by low vaccination rates in these age groups. Evidently, according to the model, the lower vaccination rates are not compensated for by infections. According to the officially reported numbers, the group of children under 5 years of age is almost entirely unvaccinated, which results in a low level of immune protection. It also becomes apparent that the different age groups were vaccinated at different times during the vaccination campaign. The elderly over 60 were vaccinated very early, so immune protection was also built up early. However, due to the early vaccination, there is already a significant decline in vaccination protection in the summer of 2021. In the younger adults, a similar but less pronounced effect is seen; the effect is barely visible for individuals under 18. Because vaccinations were no longer administered strictly by age during the winter 2021/2022 booster campaign, it can be speculated that younger individuals had a shorter interval between 2nd and 3rd vaccination than older individuals.

The simulation results show that protection varies significantly depending on the age group, the variant, the time point, and the number of administered vaccinations. In particular, there is little protection in young children; hence, according to our model, potential vaccines for this age group could have a significant effect. In addition, it is clear that a vaccine adapted to the new variants would be helpful for all ages since the mean protection in July of 2022 in all age groups is only about 50% or less.

## Discussion

This study describes a methodology that explicitly models an individual’s antibody levels and the resulting immunity to infection, using an ABM as an example framework. The incorporation of this strategy into other frameworks can facilitate the execution of complex population studies taking into account the dynamics of antibodies and their waning effects. During infectious disease outbreaks, such as the COVID-19 pandemic, the formulation of effective public health policies and interventions requires an accurate estimation of population protection and individual infection risk.

In the described use cases we emphasize the significance of incorporating antibody and waning effects into models that predict infection dynamics. If this is taken into account, a more accurate estimation of the individual risk of infection and population protection can be reached. The use cases also demonstrate the ability to infer immune protection for both individuals and populations based on variables including time, age, vaccination status, and virus variant. As a result, an enhanced understanding of immunity dynamics among disparate subpopulations can provide guidance for targeted vaccination initiatives, resource allocation, and the development of more effective public health strategies. Our model’s adaptability to other infectious diseases presents an opportunity to improve responses to future pandemics and outbreaks and increases its applicability and potential influence.

Nonetheless, our model has limitations and inherent assumptions: except for the initial immunization administered to an individual, in most cases no distinction is made between the effects of vaccinations and infections (see Sec. Method details for details). Furthermore, it is assumed that the half-life of antibodies remains the same regardless of whether they originated from infections or vaccinations. Further, we assume a correlation between antibody levels and protection against infection. This is a key aspect of many epidemiological models, including the one described in this study. This assumption is based on the understanding that the presence of specific antibodies, such as IgG antibodies, can help neutralize the virus, thereby reducing the risk of infection or the severity of the disease. For SARS-CoV-2, evidence from various studies has shown this kind of positive relationship between antibody levels and protection against infection.^5^^,^^15^^,^^16^ Research indicates that individuals with higher levels of neutralizing antibodies are generally less likely to become infected or experience severe COVID-19 symptoms. However, it is important to note that the relationship between antibody levels and protection might be non-linear. Multiple other factors, such as cellular immunity or the presence of immunological memory, might also play a significant role in determining an individual’s level of protection.

Overall, we believe that these considerations do not limit the applicability of the presented model, as the given empirical evidence from the presented use cases demonstrated. With the increasing availability of research on SARS-CoV-2, COVID-19, and other infectious diseases, it will be possible to refine and expand these assumptions to further improve the precision and applicability of our model.

In the field of infectious disease epidemiology, the methodology presented in this paper offers a valuable method for modeling antibody dynamics and protection against infection. This will help inform more effective public health policies and interventions, ultimately benefiting global efforts to combat and manage the spread of infectious diseases.

### Summary & conclusion

We have presented an approach on how to model the variant-specific neutralizing effect of antibodies and how to convert it into a protection against infection. The presented use cases demonstrate that the model produces valid results that match the observed historical data in Germany very well. Further, we have shown how this approach can be used within an agent-based modeling framework to allow computation of infection dynamics. In the (current) situation of high population immunity, considering immune protection is essential for achieving realistic simulation results.

Our model estimates that in summer 2022 there was still a significant difference in immune protection between unvaccinated and vaccinated individuals. According to the model, the lack of vaccination was not fully compensated for by infections. This effect also becomes clear when looking at the age groups: according to the model, children had a significantly lower protection against infections than adults. In addition, the model allows quantification of the protection against the immune escape variants. These results suggest that the protection against the Omicron variants is significantly lower than that against the original (wild-type) variant. This matches the available data.

The necessary model parameters have either been taken from the available literature or are based on calibration to available data. This process necessarily includes modeling choices. Given the solid agreement between our model results and the available data, we are confident that sensible parameters and fitting parameter values have been identified. This is also confirmed by simulation results that have been achieved by using the presented antibody model in conjunction with our own ABM (see, for example, Mueller et al.^17^ and Mueller et al.^18^). These results demonstrate once more that the ABM with the presented antibody-model extension is able to soundly replicate many important parameters, such as case numbers, R values, and hospitalizations. This is a significant improvement based on the explicit antibody model for each agent.

To the best of our knowledge—and based on our literature review—no other currently available model allows both (1) the integration of antibody levels as a proxy for protection against infection and (2) the modeling of individual immunization histories. While a small number of models implemented one of these, we have not encountered any that implements both. In consequence, our approach could help others to integrate any permutation of immunization events, as well as waning, into their COVID-19 models.

### Limitations of the study

The proposed model, as it is described in this paper, is well suited for the presented application scenarios and can also be adapted for future use cases. However, limitations and possible improvements exist and will be briefly described in the following.

As described in detail in the STAR Methods section, our model uses a uniform factor of 15 to increase antibodies for all vaccinations and infections, if this is not the first immunization event. That is, for this parameter, we do not distinguish between the different virus variants or vaccines. In principle, that factor could be different for each virus or vaccine variant, and/or for each prior immunization sequence. Differentiating between all these would yield many free parameters, which would be difficult or impossible to calibrate. This would go against the purpose of the model to be used for predictive simulations during the acute phase of a pandemic. For this, certain simplifications are necessary. The simplification here was to use the factor of 15 from data for the most typical case, which was a booster vaccination after the initial vaccination, and then to assume that the same factor applies for all other cases. Clearly, it would be possible during a pandemic to systematically test for other cases, for example, for a vaccination after an infection, or an infection after a vaccination, and from that to decide whether the values need to be differentiated or not. For the COVID pandemic, we were not aware of such studies.

Furthermore, when it comes to waning, we do not distinguish between infection and vaccination, but assume the same half-life. This is on the one hand due to the fact that we have not found any data that assume the opposite, and it on the other hand once more avoids that the number of model parameters increases significantly. Where possible, we have generally tried to reduce the number of model parameters.

Adjustments to the model would be straightforward since the entire source code^19^ is publicly available.

## STAR★Methods

### Key resources table

**Table:** 

REAGENT or RESOURCE	SOURCE	IDENTIFIER
**Deposited data**		

Mobility Data Cologne	This paper	https://doi.org/10.5281/zenodo.8137677

**Software and algorithms**		

EpiSim	Müller et al.^10^	https://github.com/matsim-org/matsim-episim-libs

### Resource availability

#### Lead contact

Further information and requests for source code and data should be directed to and will be fulfilled by the lead contact, Sebastian Müller (mueller@vsp.tu-berlin.de).

#### Materials availability

This study did not generate new materials.

### Experimental model and study participant details

Our study does not use experimental models typical in the life sciences.

### Method details

In the following, we describe how our model computes antibody levels and how it calculates the ensuing protection against infection with SARS-CoV-2. We explain how the model parameters were chosen using both available data and our own calibration, which was necessary to fill data gaps.

The model is composed of two layers: 1) Modelling the antibody level, based on real-world measurements of antibody titers. 2) Translation of the antibody level into protection against infection, as this is the relevant parameter to calculate the infection probability.

The model is designed as an extension to our agent-based model presented in Mueller et al.^10^ The protection is integrated as an additional parameter into the infection model, described on page 5 of that paper. The principle is simple: the higher the antibody level, the higher the protection. And the higher the protection, the lower the probability of infection, given contact with an infectious agent. As described in Mueller et al.,^10^ our dose response model is calibrated against both hospital numbers and case numbers. Among other things, disease import (e.g., from abroad), activity participation based on mobile phone data, and mask wearing are taken into account.

#### Motivation

Our approach has two motivations. *First,* around fall 2021 with the emergence of the Delta variant, it became clear that modelling immunity with look-up tables would eventually become combinatorial impossible. Already at that time, four immunization events (initial vaccination, booster, two infections, in arbitrary sequence) were not unheard of, and together with four possible types of immunization (wildtype/Alpha variant, Delta variant, mRNA vaccination, vector vaccination) this resulted in $4^{4}=256$ different immunization sequences, and that number kept growing combinatorial with each additional immunization event, virus strain, or vaccine type. It became impossible to derive immunities for all these different sequences, in particular since they also needed to be further differentiated by the timings between the immunization events. In consequence, a plausible quantitative model was needed that could be fitted against the available data, and that would extrapolate to immunization sequences for which we had no data.

*Second,* we needed the model to work with new virus strains before vaccine effectiveness data became available for those. One of the first things that became known about each new strain were the relative antibody titers: Blood samples were taken from individuals with a known immunization history, and it was measured how much these samples could be diluted until the new virus variant, compared with previous virus variants, would overwhelm the diluted blood samples. Our model is able to quantitatively predict the remaining immunity against such immune escape variants from such neutralization titer studies.

#### Background

We used the models of Cohen et al.^25^ and Cromer et al.,^46^ with details for the latter in Khoury et al.,^47^ as starting points for the process of integrating antibodies into our agent-based model. Both deal with the connection between neutralizing antibodies with protection against infection. They both postulate a logistic model of type

$$VE=\frac{1}{1+\exp\;\left(-\beta \cdot \left(\log\left(N_{ab}\right)-\log\left(N_{50}\right)\right)\right)}$$

for vaccine effectiveness, where $N_{ab}$ is the measured antibody level, $N_{50}$ is the antibody level at which VE is 50%, and β determines the slope at $N_{ab}=N_{50}$. Translated into relative risk, which we here call immFac, this can be rearranged to

$$immFac=1-VE=\frac{1}{1+\exp\;\left(\beta \cdot \left(\log\left(N_{ab}\right)-\log\left(N_{50}\right)\right)\right)}=\frac{1}{1+{\left(N_{ab}/N_{50}\right)}^{\beta }}=\frac{1}{1+N^{\beta }}\text{,}$$

where *N* is a strain-specific relative antibody level and is defined as $N:=N_{ab}/N_{50}$ (see Sec. Modelling the antibody level for more explanation). *N* is unit-less and would need to be multiplied with $N_{50}$ to be expressed in laboratory units. Note that *N* is time-dependent, as antibodies decrease over time, and increase when an infection or vaccination occurs (see Sec. Modelling the antibody level). The value for β is chosen through calibration (see also Sec. Calibration). The equation shows that a relative antibody level of 0 leads to an immunity factor of 1, i.e. a VE of 0%. An antibody level of 1 leads to an immunity factor of 0.5, i.e. a VE of 50%. An antibody level above 1 corresponds to an immunity factor below 0.5, i.e. a VE higher than 50%.

#### Integration with a dose-response model

Our agent-based model^10^ uses the following well-established dose-response model to calculate the probability of infection:^48^^,^^49^^,^^50^

$$p_{\inf}=1-\exp\;\left(-\Theta \cdot d\right)\text{,}$$

where *d* is the viral dose, and Θ is a calibration parameter, which depends on the transmissibility of the virus under consideration.

The open question was how to include immFac in the above dose-response infection model; since most simulations use a compartmental approach, they do not need to resolve this issue. A possible form, $p_{\inf}^{immunized}=immFac\cdot p_{\inf}^{not-immunized}$, would imply full protection for people with high antibody levels, even in virus rich environments.^51^ This does not seem plausible, given that the virus eventually overcomes the antibodies if the ratio of virus to antibodies is large enough.

As a consequence, we put immFac into the exponent:

(Equation 2) $$p_{\inf}^{immunized}=1-\exp\;\left(-\Theta \cdot d\cdot immFac\right)=1-\exp\;\left(-\Theta \cdot \frac{d}{1+N^{\beta }}\right)\;\text{.}$$

Note that this has the consequence that in a virus-limited environment, where dose *d* is small, immFac becomes a risk reduction:

(Equation 3) $$\frac{p_{\inf}^{immunized}}{p_{\inf}^{not-immunized}}=\frac{1-\exp\;\left(-immFac\cdot \Theta \cdot d\right)}{1-\exp\;\left(-\Theta \cdot d\right)}\approx \frac{immFac\cdot \Theta \cdot d}{\Theta \cdot d}=immFac\;\text{.}$$

This linear approximation in a virus-limited environment follows from 1−exp(−x)≈x for x≥0 and sufficiently small.

That is, a model that was originally developed for a macroscopic situation is now used at a more microscopic level. The *epidemiological* risk reduction would come out as an average over many exposures with different values of *d*.

Equation 2 shows how antibodies reduce the likelihood of becoming infected (reduced susceptibility). However, we also included the fact that individuals with antibodies have reduced probability to transmit the virus (reduced infectivity). Thus, when an unvaccinated agent has contact with a vaccinated agent, the unvaccinated agent indirectly benefits from the vaccinated agent’s antibodies because the probability of infection is reduced. If both agents are vaccinated, the probability is further reduced. This is in accordance with findings by Eyre et al.^52^ In our model, the infectivity is reduced according to the same principle as explained above, but to a lesser extent. The effect of the antibodies on infectivity is 25% of the effect they have on susceptibility. Thus, if an agent has a 50% reduced probability of infection due to their antibodies, the probability of transmission is reduced by only 12.5%.

#### Modeling the antibody level

In the next step, the relative antibody levels (*N* in Equation 2) are modelled. For every simulated day and agent, the model updates the agent’s relative antibody level with respect to each SARS-CoV-2 strain. A relative antibody level of 0 corresponds to no protection, while a relative antibody level of 1 corresponds to 50% protection (see Sec. Background). At the beginning of the simulation, all agents are initialised with a relative antibody level of 0. Immunization events (vaccinations and infections) increase an agent’s relative antibodies. On days on which no immunization event occurs, the antibody levels follow an exponential decay curve,

(Equation 4) $$N\left(t\right)=N_{0}\cdot 2^{-t/t_{0.5}}\text{,}$$

where N(t) is the antibody level on day *t* after the most recent immunization event, $N_{0}$ is the antibody level immediately after the most recent immunization event and $t_{0.5}$ is the half-life, which is calibrated as 60 days, see Sec. Calibration. The value of 60 days is on the lower end of what can be found in the literature^46^^,^^53^^,^^54^).

The general principle of the model is exemplified in Figure 4. The left figure shows how the antibody level of an agent develops over time. The spikes in relative antibodies correspond, in this illustrative example, to a SARS-CoV-2 infection, an mRNA vaccination, and an infection with the Delta variant. On days without an immunization event, the waning becomes apparent. In addition, it becomes clear that we distinguish between the different virus variants. As a result, this means that agents are less protected against the immune escape variants after vaccination. The right plot shows how we translate the antibodies into protection. See Sec. Background and Sec. Integration with a dose-response model for details.

##### Initial immunization

As noted above, we assume that initially (at the beginning of the pandemic) all agents have an antibody level of 0. The first immunization event generates a strain-dependent initial antibody level, which is shown in Table S1. The agent’s antibodies have varying neutralizing effects against different SARS-CoV-2 strains. Thus, we model that an agent has a different relative antibody level per strain. As shown in Table S1, an infection with Delta provides more relative antibodies against a reinfection with Delta than against an infection with an Omicron variant. Similarly, the vaccinations were designed to protect against the wild-type and Alpha variants; thus, the vaccinations provide more relative antibodies against these strains than against later variants.

Table S1 is based on studies that examined protection against (symptomatic) infection and on various studies that measured antibody titers after vaccination or infection.^55^^,^^56^ Here, protection obtained through vaccination with the mRNA vaccines developed by Moderna (mRNA-1273), and by BioNTech-Pfizer (BNT162b2) are summarized under ‘mRNA’, while the vector vaccines developed by AstraZeneca (ChAdOx1-S) and Johnson & Johnson (Ad26.COV2.S) are summarized under ‘vector’. In consequence, we do not distinguish between vaccine brands, but only between vaccine types.

The starting point for Table S1 was protection after vaccination with an mRNA vaccine against the wild-type, the Alpha, the Delta and the Omicron BA.1 variant (marked with ⋆ in Table S1). For these cases, studies that assess vaccine effectiveness over time are available.^15^^,^^16^^,^^21^^,^^22^^,^^23^ To match these studies, the corresponding initial antibody values in Table S1 were calibrated. In the same step, the half-life of 60 days from Equation 4 was estimated (for the calibration process, see Sec. Calibration and for the conversion between vaccine effectiveness and neutralizing antibodies, see Equation 6.

In the next step, we used measurements from Roessler et al.^55^^,^^56^ to populate the other entries.

For example, the second row of Table S1 represents the relative antibodies versus various strains resulting from a vector vaccination. For Alpha,^55^ measure a neutralizing effect of approximately 700 after mRNA vaccination and approximately 210 after vector vaccination (we obtained these values from Figure 1 in Roessler et al; ^55^). We used this ratio to calculate the relative antibodies against Alpha after vector vaccination: 29.2·210/700=8.76. The remaining entries in the table were filled following the same logic.

The measurements by Roessler et al.^55^^,^^56^ and others show that there is virtually no neutralizing effect if the initial immunization event is an Omicron infection, so we assume a very low value (0.01) here. We do not use 0, as it is to be expected that at least a small protection is present in the case of repeated infections.

We did not have accurate measurements for Omicron BA.2 and BA.5 at the time of the study; thus, we calibrated the immune escapes using our agent-based model. Here, we take the values for BA.1 from Table S1 and divide them by a factor. The factor was calibrated so that our model correctly replicates the infection dynamics, in particular the initial growth of BA.2 and BA.5, respectively.

##### Agent heterogeneity

To account for the fact that immune response towards vaccinations or infections varies across the population, we assign an *immuneResponseMultiplier* to each agent. The lowest possible *immuneResponseMultiplier* is 0.1, which is an attempt to adequately depict the immunocompromised population; the maximum multiplier is 10.0. Table S1 presents the initial antibodies for an individual with an average response to immunization events (*immuneResponseMultiplier* = 1.0); for low and high responders, the antibodies shown in the table are multiplied by an agent’s *immuneResponseMultiplier* to calculate the antibodies gained in response to an immunization event. A log-normal distribution of *immuneResponseMultiplier* with a μ of 0.0 (corresponding to a median of 1.0) and σ of 3.0 is applied to the population.

##### Subsequent immunizations

If the agent is subject to an additional immunization event, their antibody levels against each strain will be multiplied by a factor of 15, regardless of whether a vaccination or infection occurs.^57^ The maximum antibody level that an agent can have is 150 (which corresponds to a protection of nearly 100%). If the multiplication by 15 still leads to a lower protection than indicated in Table S1, then the value from Table S1 is used insead. This means that, at minimum, the initial antibody level from Table S1 is always reached.

#### Calibration

As not all necessary parameters were available in the literature when we built this model, some had to be estimated. This is also consistent with using the model in real time while the pandemic is unfolding. These estimations were based on studies on vaccine effectiveness, together with Equations 2 and 4. The relative risk for a symptomatic infection of an immunized individual vs. a non-immunized individual given dose *d* is $p_{symp}^{immunized}/p_{symp}^{not-immunized}$. Vaccine effectiveness is defined as one minus this relative risk:

(Equation 5) $$VE=1-\frac{p_{symp}^{immunized}}{p_{symp}^{not-immunized}}$$

In the model of Mueller et al.,^10^ $p_{symp}=\alpha \cdot p_{\inf}$ (with α=0.8), and thus

(Equation 6) $$\ldots =1-\frac{\alpha \cdot p_{\inf}^{immunized}}{\alpha \cdot p_{\inf}^{not-immunized}}=1-\frac{1-\exp\left(-\Theta \cdot \frac{d}{1+N^{\beta }}\right)}{1-\exp\left(-\Theta \cdot \;d\right)}\text{,}$$

that is, for the specific model of Mueller et al.,^10^ the α cancels out. The approach will, however, also work for other models where this is not the case. VE, according to Equation 6, depends on the dose *d*; for example, for d→0 one obtains $\to 1-\frac{1}{1+N^{\beta }}$ , while for d→∞, one obtains VE→0. That is, according to the model, immunity can be overcome by a sufficiently high dose. This is similar to the distinction between virus-rich and virus-limited environments, where protection measures such as masks only make a difference in virus-limited environments.^51^ We performed the calibration assuming a value of Θ·d=0.001, that is, with

(Equation 7) $$VE\left(t\right)=1-\frac{1-\exp\;\left(-0.001\cdot \frac{1}{1+N{\left(t\right)}^{\beta }}\right)}{1-\exp\;\left(-0.001\right)}\text{.}$$

We also tested the calibration for values other than 0.001 and obtained very similar results. In the model of Mueller et al.,^10^ a value of 0.001 corresponds to contact with a contagious person for about 1000sec without protection (e.g., mask) in a room of $20m^{2}$.(A typical value for Θ in the model of Mueller et al.^10^ is of the order of $10^{-5}$. At the same time, without protection (e.g., masks) d=τ/(rs·ae), where τ is the time of exposure in seconds, rs is room size in $m^{2}$, and ae is the air exchange rate per hour. Assume $rs=20m^{2}$ and ae=0.5/h, typical values for a two-person office or a smallish living room, and τ=1000sec of exposure time. These values result in Θ·d=0.001.).

Using the above equation, we performed parameter estimations for:

• $t_{0.5}$ in Equation 4.
• Entries marked with ⋆ in Table S1.
• β in Equation 2.

To this end, for the wild-type/Alpha, the Delta, and the Omicron BA.1 variants, Equation 7 was fitted to data points taken from studies^15^^,^^16^^,^^21^^,^^22^^,^^23^ by minimizing the mean squared error. For this, we used R (version 4.1.1) and the optim function from the stats package.^58^ Optim can be used for general purpose optimization as it is based on Nelder–Mead, quasi-Newton and conjugate-gradient algorithms. The results can be seen in Figure 5, where the dots are values taken from the studies mentioned above, and the lines show the vaccine effectiveness VE(t) in our model when using the calibrated values. The increased vaccine protection after 210 days is related to booster vaccinations.

#### Use cases

##### Use case 1

Use case 1 shows how our model can be used to calculate population-wide immunization statistics for a synthetic population. For this, we used the agent-based model described in Mueller et al.^10^ in combination with the antibody model introduced in this paper. We start the simulation in February 2020. At the beginning of the simulation, the population does not have any antibodies. This means that the number of immunization events is 0 for every synthetic person. In the course of the simulation, agents get infected and vaccinated and thus develop antibodies and thus protection against reinfection. For the comparison in Table 1 we analyzed which share of the synthetic population has had at least one infection or vaccination and which share of the synthetic population has had the one infection independent of the vaccination status. For the comparison in Figure 1 we analyzed the share of the population that has had at least one infection or vaccination at various different time points. The model data presented in Table 1 refers to the whole adult population (ages 18+). The model data presented in Figure 1 refers to the whole population. All values are mean values for the respective group.

##### Use case 2

The results shown in the second use case are based on the same simulations as the first use case. Since we record the antibodies for each synthetic person for each simulated day, we can aggregate these data to the plots shown. Since we also record when each person is vaccinated, we can calculate the antibodies separately by vaccination status. For the analysis, we use one data point per month to save computing time during the analysis and to keep the figures clear. However, the actual simulation runs on each day, so that a day-by-day view would be possible.

#### Antibody models for epidemiological predictive modeling of COVID-19: A literature search

The previous section described our approach for modelling antibody levels in an epidemiological context. In this section, we present an overview of similar approaches that exist in the literature, as of July 2022. We compiled a list of all models that have been listed in one of the following resources: (a) the *Covid-19 Forecast Hub* (From the community sub-section, as of 07/21/22.) (^59^), (b) the *European Covid-19 Forecast Hub* (From the community sub-section, as of 07/21/22.) (^60^^,^^61^), and (c) the *European Covid-19 Scenario Hub* (From the models sub-section, as of 08/31/22.) (^62^). The final list contains 90 models. Additionally, to the 86 models from the three resources, we also included four more models that we found through a PubMed literature search. The full list can be found in Appendix A. To get the relevant information for the individual models, we went to the respective websites, and analyzed connected publications and available source codes (e.g. from GitHub). We were in particular interested in models that either (1) related antibodies and protection against infection and integrated this into their model or (2) acknowledged and integrated into their model the waning of protection against infection (after vaccination and/or infection).

From this literature review, we conclude that, apart from Covasim,^24^^,^^25^ whose influence on our model is discussed in Sec. Background and which can be found as model # 1 in Appendix A, none of the reviewed models explicitly integrates antibody levels as part of their infection sub-model. This is also due to the fact that many models focus on the prediction of hospitalization numbers and thus do not need to explicitly model individual antibody levels. However, some approaches seem to have integrated some kind of vaccination or antibody sub-model, but no detailed description was found. This includes (see Appendix A for details): IHME, UC3M-EpiGraph, ECDC-CM_ONE, and SIMID-SCM.

## Figures and Tables

**Table:** Models, which were discovered through (a) the Covid-19 Forecast Hub, (b) the European Covid-19 Forecast Hub, (c) the European Covid-19 Scenario Hub, or (d) a literature search

#	Model	EMI	Source	Model description
1	Covasim	YES	Lit. Search	Agent-based model that simulates the transmission of COVID-19. Individual may pass through the following infection stages: susceptible, exposed, infectious, and recovered (SEIR). The associated GitHub repository has last been committed to in January 2022, and their paper,^24^ which was published in July 2021, mentions that they will incorporate waning efficacy, but no more recent information or model description could be found. Their additional methods preprint,^25^ which does not explicitly mention Covasim but is noted on their project website, served as the basis for our antibody model and is discussed in Sec. 4.
2	CovidSim	N/A	Lit. Search	Agent-based model developed by MRC Center for Global Infectious Disease Analysis hosted at Imperial College, London. Model documentation, as part of the associated GitHub repository,^26^ has last been updated in February 2021. No mentions of vaccines, waning or antibodies.
3	CoSim	N/A	Lit. Search	Expanded SEIR model containing 27 compartments (as of December 2021).^27^ COVID-19-related metrics are computed on German federal state level. Checking both the model description on their website as well as the FAQs for their simulator (which have last been updated in December 2021), no information on waning or antibodies could be acquired.
4	OpenCOVID	YES	Lit. Search	A stochastic, discrete-time, individual-based transmission model of infections and disease dynamics. In their paper,^28^ dating from December 2021, the authors note that they did not consider waning immunity in this study. But, in the supplemental information, they model the probability of transmission as $p\left(transmission\right)=\beta \cdot \nu_{i}\left(\tau \right)\cdot \varphi_{1}\cdot \sigma \left(t\right)\cdot \left(1-\mu_{s}\right)$, where $\nu_{1}\left(t\right)\in \left[0,1\right]$ denotes the viral load of the infectious individual and τ denotes they days following infection. $\varphi_{1}$ denotes the infectivity factor of the SARS-CoV-2 variant with which the infectious individual is infected, σ(t) denotes the season factor at date *t*, and $\mu_{s}$ denotes the immunity of the susceptible individual. Here, $\mu_{s}=83\%$ for recovered individuals (independent of disease severity, risk group or age) and $\mu_{s}=80\%$ for vaccinated individuals. GitHub repository has last been committed to in January 2022. Hence, we could not determine whether or not they are still continuing their work and if they have by now integrated waning immunity into their model.
5	KITmetriclab	NO	Covid-19 Forecast Hub	Models from the COVID-19 Forecast hub are ranked according to their performance over the previous four weeks and then ensembled and weighted iteratively to achieve a combined forecast.
6	CovidAnalytics at MIT/MIT_CovidAnalytics-DELPHI	N/A	Covid-19 Forecast Hub, European Covid-19 Forecast Hub	Expanded SEIR model. Their technical report^29^ was published in July 2020 and consequently does not contain vaccinations, waning, or antibodies.
7	UMass-Amherst	N/A	Covid-19 Forecast Hub	Model is based on the “HHS Protect daily Covid-19 hospital’ admission data. Creating a set of simple time-series baseline models, which are then combined into a single ensemble forecast of hospitalizations. No documentation provided.
8	Johns Hopkins University Applied Physics Lab – Bucky	NO	Covid-19 Forecast Hub	Age-stratified SEIR model to estimate mid-term case loads and provide additional outputs relating to the associated healthcare burden on county level. No mention of waning immunity and antibodies could be found.
9	MOBS Lab at Northeastern	NO	Covid-19 Forecast Hub	Usage of the Global Epidemic and Mobility (GLEAM) model, an individual-based, stochastic, and spatial epidemic model. Four week forecast of weekly hospitalizations and deaths and US state and national level. Projections have not been updated since April 2022, no mention of waning or antibodies.
10	Masaryk University/MUNI-ARIMA/MUNI-LaggedRegARGIMA/MUNI-Var	N/A	Covid-19 Forecast Hub, European Covid-19 Forecast Hub	Newest forecasts on their website are for late 2021/early 2022. They claim that methods of statistical time series analysis are used, but no further explanation is provided.
11	GT	NO	Covid-19 Forecast Hub	Deep learning model that is data driven and learns the dependence of hospitalization and mortality rate based on a variety of syndromic, demographic, mobility, and clinical data. Their preprint^30^ from March 2021 presents their model in detail but does not mention vaccinations, waning immunity, or antibodies.
12	COVID-19 Forecast Hub	N/A	Covid-19 Forecast Hub	Baseline predictive model to forecast number of cases. No documentation provided on website or associated GitHub repository.
13	HKUST	NO	Covid-19 Forecast Hub	Deep neural networks to forecast cumulative deaths on the US state level. Here, deaths, cases, and hospitalization data are taken into account.
14	QJHong	NO	Covid-19 Forecast Hub	“Encounter Density” (which is based on cell phone data) is used to predict the future reproduction number and confirmed cases.
15	Predictive Science Inc	N/A	Covid-19 Forecast Hub	Associated GitHub repository provides no documentation. Hence, no information about the model could be acquired.
16	IDSS COVID-19 Collaboration (Isolat) at MIT	NO	Covid-19 Forecast Hub	Curve-fitting model to make short-term (for the following two weeks) predictions on number of cases and deaths in the US, on state and federal level. Based on the assumption that the metric of interest (i.e., the number of deaths) can be explained by the sum of a set of Gaussian curves.
17	Steve McConnell	N/A	Covid-19 Forecast Hub	Forecasts deaths in the US; work is submitted to CDC to integrate into their ensemble model. No description of model provided.
18	Robert Walraven/RobertWalraven-ESG	NO	Covid-19 Forecast Hub, European Covid-19 Forecast Hub	Website has not been updated since 2021. According to the author, no epidemiological parameters are used. Contrarily, he uses a skewed Gaussian distribution to fit the available case data and a second skewed Gaussian distribution to fit the available deaths data.
19	Carnegie Mellon Delphi Group (COVIDcast)	NO	Covid-19 Forecast Hub	Creation and evaluation of an ensemble forecast.
20	Predictive Science	N/A	Covid-19 Forecast Hub	The associated GitHub repository states that this is a “R package for modeling and forecasting direct-contact and vector-borne infectious diseases”. No further documentation provided.
21	UT	N/A	Covid-19 Forecast Hub	Consortiuum, which both surveils and forecasts the infection dynamics of COVID. In their paper from February 2022 they present their age- and risk-structured SEIR model and note the necessity for forecasting models to integrate dynamics of infection-acquired and vaccine-acquired immunity. At the time of our screening, no publications nor documentation on the website which included acquired immunities, vaccines, or antibodies could be found.
22	LUcompUncertLab	N/A	Covid-19 Forecast Hub	Associated GitHub repository states that they forecast infectious disease dynamics by combining forecasts of computational models and human judgment. No documentation provided.
23	UCSD_NEU	NO	Covid-19 Forecast Hub	A hybrid mechanistic and deep learning model for short-term (up to 4 weeks ahead) predictions of deaths on US state level.
24	University of Virginia, Biocomplexity COVID-19 Response Team/UVA-Ensemble/UVA-EpiHiper	N/A	Covid-19 Forecast Hub, European Covid-19 Forecast Hub, European Covid-19 Scenario Hub	Multi-method model (integrating multiple statistical, machine learning, and mechanistic methods) forecasting the new confirmed cases on US state, county, and national level. These model forecasts are combined using Bayesian model averaging.^31^ In the publication from August 2021, neither antibodies nor waning are mentioned.
25	MIT-Cassandra	N/A	Covid-19 Forecast Hub	Associated GitHub states that this is a Markov decision process inference model to capture the dynamics of the growth rates of cases and deaths. No documentation provided.
26	University of Central Florida	N/A	Covid-19 Forecast Hub	The associated GitHub repository contains a variety of CSV files, but no documentation. Hence, no information on the model could be gathered.
27	Columbia University	N/A	Covid-19 Forecast Hub	Use a metapopulation SEIR model to forecast for the upcoming 42 days of daily new cases, infections, and hospitalized individuals on US state, county, and national level. The model documentation, which we scanned, dates from 2020 and hence does not mention antibodies or waning. No more recent documentation was found.
28	Hussain Lab at Texas Tech University	N/A	Covid-19 Forecast Hub	Associated GitHub repository states that this is an SIR-based compartmental model, which takes into account the possible immunity loss for recovered individuals. No documentation provided and linked preprint is from July 2020. Hence, this does not contain any valuable information for this study.
29	CU Boulder	NO	Covid-19 Forecast Hub	Associated GitHub repository states that they predict COVID-19 cases at the county level in the US using a stacked long short-term memory model (LSTM). In their paper,^32^ neither antibody levels or waning are mentioned as a necessity for their model. Same group as in ‘CUB_PopCouncil’ below.
30	’Swiss Data Science Center/University of Geneva’/SDSC_ISG-TrendModel	NO	Covid-19 Forecast Hub, European forecast hub	Short-term (1 week ahead) predictions of cases and deaths; very little documentation provided on website. In their preprint, they clarify that they implemented a piecewise trend estimation method based on the robust Seasonal Trend decomposition procedure based on LOESS (STL).
31	Karlen Working Group	N/A	Covid-19 Forecast Hub, European Covid-19 Forecast Hub	Usage of discrete-time difference equations with long periods of constant transmission rate. Their model description from July 2020 does not mention waning or immunity, but they provide some presentation slides, which mention the integration of waning into their model. No further documentation is provided and antibodies are not mentioned.
32	University of Southern California/USC-SlkJalpha/USC-SlkJalpha_update	N/A	Covid-19 Forecast Hub, European Covid-19 Forecast Hub, European Covid-19 Scenario Hub’	Usage of their own SIkJα model to forecast cumulative cases on US state, county, and national level as well as internationally. Their preprint^33^ dating from July 2020 does not mention antibodies or waning, but their website notes that they are accounting for vaccines and all current variants. Here, no further documentation could be found.
33	CUB_PopCouncil	NO	Covid-19 Forecast Hub	Associated GitHub repository provides very little documentation. According to the repository, this is code for predicting COVID-19 hospitalizations at the state level in the US. Hereby, a stacked long short-term memory model (LSTM) is used.
34	John Hopkins ID Dynamics COVID-19 Working Group	N/A	Covid-19 Forecast Hub	SEIR model incorporating the uncertainty in the effectiveness of NPIs to project different possible epidemic trajectories and healthcare impacts. Associated GitHub repository has last been updated in September 2020, and paper^34^ dates from April 2021. Paper does not yet consider vaccines and mentions neither waning nor antibodies. No more recent documentation could be acquired.
35	BPagano	N/A	Covid-19 Forecast Hub	Death-based SIR model to project cumulative confirmed cases, confirmed cases per day, cumulative deaths, deaths per day, and some additional metrics for a variety of individual countries and on US state and county level. Neither antibodies nor waning immunity are mentioned in the model description.
36	Institute for Health Metric and Evaluation (IHME)	YES	Covid-19 Forecast Hub	(0) Blog post dating from December 2021,^35^ which describes their model update in detail. Their new model is a system of integro-differential equations. (1) Individuals are placed in different compartments based on variant by which they were more recently infected and round of vaccination they most recently got → One compartment for each combination of vaccinations and infections (at the time of the blog post they are considering 24 compartments); (2) Time since last vaccination and/or infection is tracked; (3) Protection from infection and vaccine interact multiplicatively. In other words, if ε is the protection (against a variant) acquired from vaccination and φ is the protection acquired from infection, then a person’s risk of infection is (1−ε)(1−φ) times the risk of a naive individual. Hence, an individual’s risk of infection depends on the variant and time of their last infection, the brand and time of their last vaccination, and the variant they’re currently confronted with. They estimate the average protection in a particular susceptible compartment to a specific variant. This is then integrated into their integro-differential equations which describe the transitions between compartments. In the appendix of their recent publication^36^ it is noted that to estimate waning protection against infection following vaccination, they used Bayesian meta-regression with a monotonically decreasing spline on time since second dose. Waning curves are estimated by vaccine and a lower bound of a 10% efficacy is introduced.
37	BIOCOMSC-Gompertz	N/A	European Covid-19 Forecast Hub	Description of their (early) empirical model can be found in their report from March 2020. Hence, no mention of partial (immunity), antibodies, or waning. Unfortunately, large parts of the website are in Catalan, so they were not considered in this review.
38	bisop-seirfilter	NO	European Covid-19 Forecast Hub	Unfortunately, their website is only available in Czech. However, in their preprint^37^ from February 2021 they describe their SEIR compartmental model. Neither immune waning nor antibodies are mentioned.
39	bisop-seirfilterlite	NO	European Covid-19 Forecast Hub	Same people as for ‘bisop-seirfilter’.
40	CovidMetrics-epiBATS	N/A	European Covid-19 Forecast Hub	Short-term (for the following 10 days) predictions of cases for Germany and its federal states. Apart from that, visualizations of different metrics, no documentation provided.
41	DirkBeckmann-Gompertz	N/A	European Covid-19 Forecast Hub	Accessed the associated GitHub repository, which did not provide any documentation. Hence, no documentation about the model could be acquired.
42	DSMPG-bayes	N/A	European Covid-19 Forecast Hub	Bayesian inference and forecast of different COVID-19-related metrics like the effective growth rate, daily new reported cases, and the total of reported cases.
43	ECDC-hosp_model	N/A	European Covid-19 Forecast Hub	Ensemble model forecasts by the European COVID-19 forecast hub predict the numbers of cases, hospital admissions, and deaths to be reported for the upcoming two weeks for every EU country. ECDC’s forecasts of ICU admissions are currently not displayed as the model is undergoing adjustments. Apart from description of data sources, no documentation could be found.
44	epiforecasts-arimareg	NO	European Covid-19 Forecast Hub	Estimate and forecast effective reproduction number and confirmed cases based on case and death notifications while accounting for reporting delays.
45	epiforecasts-caseconv	NO	European Covid-19 Forecast Hub	Leads to the same website as ‘epiforecasts-arimareg’.
46	epiforecasts-EpiExpert_direct	NO	European Covid-19 Forecast Hub	Leads to the same website as ‘epiforecasts-arimareg’.
47	epiforecasts-EpiExpert_Rt	NO	European Covid-19 Forecast Hub	Leads to the same website as ‘epiforecasts-arimareg’.
48	epiforecasts-EpiExpert	NO	European Covid-19 Forecast Hub	Leads to the same website as ‘epiforecasts-arimareg’.
49	epiforecasts-EpiNow2	NO	European Covid-19 Forecast Hub	Package to estimate the effective reproduction number, growth rate, and doubling time.
50	epiforecasts-tsensemble	NO	European Covid-19 Forecast Hub	Leads to the same website as ‘epiforecasts-arimareg’.
51	epiforecasts-weeklygrowth	N/A	European Covid-19 Forecast Hub	Leads to the personal website of Sam Abott, who is also involved in the models mentioned above. No model documentation could be acquired.
52	epiMOX-SUIHTER	N/A	European Covid-19 Forecast Hub	Depicts data of the COVID-19 pandemic at the national Italian as well as on a regional (20 Italian regions) level. Their paper^38^ from July 2021 mentions neither antibodies nor immune waning but rather evaluates the ability of their dashboard to provide fast and in-depth analyses of the past trends of the pandemic in Italy and supply predictions on its evolution based on their compartmental model, named SUIHTER.
53	EuroCOVIDhub-baseline	N/A	European Covid-19 Forecast Hub	Forecasts and reports can be found on the European Covid-19 Forecast Hub website, but no documentation could be found.
54	EuroCOVIDhub-ensemble	N/A	European Covid-19 Forecast Hub	Same as above.
55	FIAS_FZJ.Epi1Ger	N/A	European Covid-19 Forecast Hub	Broken URL, no information could be acquired.
56	fohm-c19inbel	N/A	European Covid-19 Forecast Hub	Broken URL, no information could be acquired.
57	HZI-AgeExtendedSEIR	N/A	European Covid-19 Forecast Hub	They are currently conducting a multi-local and serial cross-sectional prevalence study on antibodies in Germany, but no model (description) on infection dynamics could be found on their website.
58	ICM-agentModel	N/A	European Covid-19 Forecast Hub, European Covid-19 Scenario Hub	Predict confirmed cases, occupied hospital beds, and critical cases for the following two months in Poland. Agent-based model, but hardly any model documentation provided on website. Their preprint^39^ from September 2021 does mention neither antibodies nor waning, describes their agent-based model (not mentioning the influence of multiple vaccine jabs/infections), and focuses on finding an optimal lockdown strategy for Poland. In said preprint, they define the probability of infection as $p_{infection}=1-\exp\left(-\alpha I\right)$. Here, α is the transmission coefficient and *I* is the total infectivity defined via $I=\sum_{c}w_{c}I_{c}$, where the sum is over the different infection contexts (household, workplace, preschool, school, university, large university, street, and travel), $w_{c}$ is the time-dependent contact rate of context *c*, and $I_{c}$ is the time-dependent context infectivity depending on the number of (a) symptomatic and all agents at context *c*, the fraction of symptomatic agents who do not self-isolate, and a curbing parameter for the infectivity of the asymptomatic agents. Consequently, no protection acquired from infection/vaccination is included.
59	IEM-Health-CovidProject	N/A	European Covid-19 Forecast Hub	AI-based disease model to predict the number of new cases during the following seven days in the USA. Dashboard has not been updated since April 2021, no model description available.
60	ILM-EKF	N/A	European Covid-19 Forecast Hub	Leads to a GitHub user page on which no documentation is provided. Unable to acquire information.
61	Imperial-DeCa	NO	European Covid-19 Forecast Hub	Short-term forecasts of COVID-19 deaths in multiple countries. Produce ensemble forecasts from the output of three different models.
62	Imperial-Rtl0	NO	European Covid-19 Forecast Hub	Leads to same website as ‘Imperial-DeCa’.
63	Imperial-sbkp	NO	European Covid-19 Forecast Hub	Leads to same website as ‘Imperial-DeCa’.
64	itwm-dSEIR	N/A	European Covid-19 Forecast Hub	Link provided on the European COVID-19 Forecast Hub leads to the general website of Fraunhofer-Institut für Techno- und Wirtschaftsmathematik ITWM. There, no information on any sort of COVID-19 related model could be acquired.
65	ITWW-county_repro	N/A	European Covid-19 Forecast Hub	Leads to same GitHub user page as ‘ILM-EKF’.
66	JBUD-HMXK	N/A	European Covid-19 Forecast Hub, European Covid-19 Scenario Hub	Projections for various scenarios and countries. “News” subpage has not been updated since December 2021, no documentation provided. Associated GitHub repository has last been updated in April 2022 but does not provide documentation either.
67	KITmetricslab-bivar_branching	N/A	European Covid-19 Forecast Hub	GitHub repository simply states that this is a simple model of a branching process for delta and non-delta cases in Germany. No additional documentation available.
68	LANL-GrowthRate	NO	European Covid-19 Forecast Hub	Their model description (dating from October 2020) introduces their model COFFEE, which is a probabilistic model that forecasts daily reported cases and deaths. Additionally, the top of their website states that they made their last real-time forecast in September 2021. Hence, we assume that they discontinued their work. No mention of antibodies or waning.
69	LeipzigIMISE-SECIR	N/A	European Covid-19 Forecast Hub	Forward to a GitHub repo which does not provide any documentation and has last been committed to in April 21.
70	MIMUW-StochSEIR	N/A	European Covid-19 Forecast Hub	Unfortunately, their website is only available in Polish and has last been updated in July 2021. Hence, no information could be acquired.
71	MOCOS-agent1	N/A	European Covid-19 Forecast Hub, European Covid-19 Scenario Hub	Short-term forecasts (up to one month) of cases and deaths in Poland. Both their reports and linked papers are from 2020. No model documentation in English could be found.
72	MUNI_DMS_SEIAR	N/A	European Covid-19 Forecast Hub	Unfortunately, their website is only available in Czech. Hence, no information could be acquired.
73	PL_GRedlarski-DistrictsSum	N/A	European Covid-19 Forecast Hub	Unfortunately, their website is only available in Polish. No information could be acquired.
74	prolix-euclidean	NO	European Covid-19 Forecast Hub	Unfortunately, the website is mostly in French and has last been updated in May 2022. Their preprint,^40^ dating from April 2021, states that they forecast the number of ICU patients in France. No mention of waning or antibodies.
75	Statgroup19-richards	NO	European Covid-19 Forecast Hub	Unfortunately, website is only available in Italian. Associated blog is in English but has not been updated since September 2021. Their paper^41^ was published in July 2021 and introduces their parametric regression model, which is motivated by incidence indicators such as the number of hospitalized, deceased, recovered, and isolated cases. No mention of (partial) immunity, waning, or antibodies.
76	Statgroup19-spatialrichards	NO	European Covid-19 Forecast Hub	Leads to same website as Statgroup19-richards above.
77	UB-BSLCoV	N/A	European Covid-19 Forecast Hub	Broken URL, no information could be acquired.
78	UC3M-EpiGraph	N/A	European Covid-19 Forecast Hub, European Covid-19 Scenario Hub	Agent-based model, whose infection model is a SEIR++ model with 17 different compartments. A different R value is associated with every compartment. The compartments for the different stages of infection differentiate between vaccinated and unvaccinated individuals. Additionally, their vaccination model considers a decrease in protection as well as an age-dependent efficacy and the risk of vaccine failure. How these properties are incorporated is not described.
79	LZF-SEIRC19SI	N/A	European Covid-19 Forecast Hub	Unfortunately, their website is solely available in Slovenian. Hence, no information could be acquired.
80	UMass-MechBayes	N/A	European Covid-19 Forecast Hub	GitHub repository has not been updated since July 2021. Apart from the repository, there is no documentation available.
81	UMass-SemiMech	N/A	European Covid-19 Forecast Hub	Same repository as for ‘UMass-MechBayes’ is provided.
82	UNED-PreCoV2	N/A	European Covid-19 Forecast Hub	Unfortunately, the website is only available in Spanish. They note that in late March 2022 a new strategy for the surveillance and control of COVID-19 as implemented. As a consequence, the daily data that feeds into their model are no longer available and they discontinued their work. Due to the language barrier, no model documentation could be acquired.
83	UNIPV-BayesINGARCHX	NO	European Covid-19 Forecast Hub	Developed the “COVID Atlas”, which provides visual analytics on several aspects of the pandemic. Here, health (i.e., data regarding the pandemic progression), socio-economic, and socio-political data are integrated. The atlas lets the user visualize data on multiple layers but does not contain its own disease digression model.
84	UpgUmibUsi-MultiBayes	NO	European Covid-19 Forecast Hub	GitHub repository has not been updated since January 2021. In their paper^42^ from August 2021, their Bayesian multinomial and Dirichlet-multinomial autoregressive models are proposed. Here, time series of numbers of patients in exclusive categories (for example, hospitalized in regular wards, in ICU units, deceased) are estimated. No mention of (partial) immunity, antibodies, or waning.
85	USyd-OneModelMan	N/A	European Covid-19 Forecast Hub	GitHub repository has last been committed to in April 21. Use global linear models to reproduce forecasts on COVID-19 daily cases. No documentation provided.
86	ECDC-CM_ONE	N/A	European Covid-19 Scenario Hub	From the GitHub repository of the Scenario Hub we learned that they integrate that protection against infection wanes based on decaying antibody titers. No detailed model description or publications associated with this model were found. Hence, we are unable to discuss the antibody integration in this model
87	MODUS_Covid-episim	YES	European Covid-19 Scenario Hub	Our own project.
88	RIVM-vacamole	NO	European Covid-19 Scenario Hub	GitHub repository states that this deterministic, age-structured, and extended (severe disease outcomes, vaccinations) SEIR model was developed to investigate different vaccination strategies. No mention of antibodies or waning in documentation.
89	SIMID-SCM	N/A	European Covid-19 Scenario Hub	Their paper from June 2021^43^ introduces their stochastic age-structured discrete time compartmental model to describe the transmission of COVID-19 in Belgium. In their technical report from September 2022^44^ the SIMID consortium notes a constant waning rate of 1/240days based on the assumptions of the European Scenario Hub. No additional information is provided; antibodies are not mentioned.
90	TUWien-AustrianCoVABM	NO	European Covid-19 Scenario Hub	Their paper from May 2021^45^ introduces their stochastic agent-based model, which evaluates contact tracing policies in 2020. Hence, (partial) immunity is of no concern and neither waning nor antibodies are mentioned.

(a) and (b) were accessed on 07/21/22, while (c) was accessed on 08/31/22.

## Data Availability

• The cologne mobility data have been deposited at Zenodo and are publicly available as of the date of publication (see key resources table). • All original code is deposited in a Github repository and is publicly available as of the date of publication (see key resources table). • Any additional information required to reanalyze the data reported in this paper is available from the lead contact upon request.

## A Appendix

### A.1 Literature review

EMI: (YES, NO, N/A) Explicit Modeling of immunisation events (distinguish between infection, immunisation etc.) AND/OR of waning AND/OR of a relation between antibodies and protection against infection. If the model description solely mentions any of the criterion above, but provides no details, we still set EMI = N/A. If the same model was, under a slightly different name, part of multiple of our sources, we put down all names separated by a “/”.
