# Supplementary material for: Explicit modeling of antibody levels for infectious disease simulations in the context of SARS-CoV-2
Source: iScience. 2023 Aug 8;26(9):107554. doi: 10.1016/j.isci.2023.107554 (PMC10466916; doi:10.1016/j.isci.2023.107554)
Supplement: Document S1. Table S1 [file mmc1.pdf]

iScience, Volume *26*

Supplemental information

Explicit modeling of antibody levels for infectious disease simulations in the context of SARS-CoV-2

Sebastian A. Müller, Sydney Paltra, Jakob Rehmann, Kai Nagel, and Tim O.F. Conrad

Table S1: Initial relative antibodies per variant after certain immunization events, Related to STAR Methods

	initial relative antibodies against			
immunization event	SARS_CoV_2	Alpha	Delta	BA.1
mRNA	29.20*	29.20*	10.90*	1.90*
vector	8.76	8.76	5.45	0.38
SARS_CoV_2	12.5	12.5	2.33	0.57
Alpha	12.5	12.5	2.33	0.57
Delta	8.76	8.76	16.4	0.76
BA.1	0.01	0.01	0.03	0.21

Based on calibration ($\star$, see Sec. Calibration in STAR Methods for details) and Roessler et al.[1,2]. 'mRNA' means a vaccination with an mRNA vaccine (either mRNA-1273 or BNT162b2), 'vector' means a vaccination with a vector vaccine (either ChAdOx1-S or Ad26.COV2.S), and 'SARS_CoV_2/Alpha/Delta/BA.1' means an infection with the 'SARS_CoV_2/Alpha/Delta/Omicron BA.1' variant.

References

1. Rössler, A., Riepler, L., Bante, D., von Laer, D., and Kimpel, J. (2022). SARS-CoV-2 Omicron Variant Neutralization in Serum from Vaccinated and Convalescent Persons. *New England Journal of Medicine* 386.7, 698–700.
2. Rössler, A., Knabl, L., von Laer, D., and Kimpel, J. (2022). Neutralization Profile after Recovery from SARS-CoV-2 Omicron Infection. *New England Journal of Medicine* 386.18, 1764–1766.
